# Epidemiological investigation of foot-and-mouth disease outbreaks in a Vietnamese bear rescue centre

**DOI:** 10.3389/fvets.2024.1389029

**Published:** 2024-06-17

**Authors:** Anna B. Ludi, Hannah Baker, Rachel Sanki, Rosanne M. F. De Jong, Julie Maryan, Martin Walker, Donald P. King, Simon Gubbins, Georgina Limon, Kirsty Officer

**Affiliations:** ^1^The Pirbright Institute, Pirbright, United Kingdom; ^2^Animals Asia Foundation, Hanoi, Vietnam; ^3^Veterinary Epidemiology, Economics and Public Health Group, Department of Pathobiology and Population Sciences, WOAH Collaborating Centre in Risk Analysis and Modelling, Royal Veterinary College, University of London, London, United Kingdom; ^4^Department of Infectious Disease Epidemiology, Imperial College, London, United Kingdom; ^5^School of Veterinary Medicine, Murdoch University, Murdoch, WA, Australia

**Keywords:** foot-and-mouth disease, epidemiology, outbreak investigation, Asiatic black bear, Malayan sun bear, rescue centre

## Abstract

Foot-and-mouth disease (FMD) outbreaks affecting Asiatic black bears (*Ursus thibetanus*) and a Malayan sun bear (*Helarctos malayanus*) were previously reported in 2011 in two housing facilities at a Vietnamese bear rescue centre. In this study, demographic data of all animals housed in the centre at the time of the outbreaks (*n* = 79) were collected. Blood samples drawn from 23 bears at different timepoints were tested for FMDV-specific antibodies targeting using a non-structural protein (NSP) ELISA and by virus neutralisation test (VNT). The relationship between seroconversion and clinical signs was explored and epidemic curves and transmission diagrams were generated for each outbreak, where FMD cases were defined as animals showing FMD clinical signs. Outbreak-specific attack rates were 18.75 and 77.77%, with corresponding basic reproduction numbers of 1.11 and 1.92, for the first and second outbreaks, respectively. Analyses of risk factors showed that after adjusting for sex there was strong evidence for a decrease in odds of showing clinical signs per year of age. All samples collected from bears before the outbreak tested negative to NSP and VNT. All cases tested positive to VNT following onset of clinical signs and remained positive during the rest of the follow up period, while only 6 out of 17 cases tested positive to NSP after developing clinical signs. Six animals without clinical signs were tested post outbreaks; five seroconverted using VNT and three animals were seropositive using NSP ELISA. This study provides initial epidemiological parameters of FMD in captive bears, showing that FMDV is easily spread between bears in close proximity and can cause clinical and subclinical disease, both of which appear to induce rapid and long-lasting immunity.

## 1 Introduction

Foot-and-mouth disease (FMD) is a highly contagious, acute viral disease typically affecting cloven-hoofed livestock. FMD follows infection with foot-and-mouth disease virus (FMDV; family *Picornaviridae*, genus *Aphthovirus*) and is clinically characterised by fever and vesicular lesions in the mouth and on the lips, teats and feet (1). FMDV has seven known serotypes (A, O, C, SAT1, SAT2, SAT3, and Asia1) which are endemic to different regions of the world (2). The features of FMDV, including a broad host range, high transmissibility, subclinical persistence and environmental stability contribute to the transboundary threats posed by FMD and highlight the importance of international efforts to control the disease to facilitate trade (3).

Despite all cloven-hoofed animals (order *Artiodactyla*) being susceptible to FMDV, cloven-hoofed domestic livestock species, such as cattle, sheep, goats and pigs, play the most significant role in the epidemiology of FMD due to the intra- and inter-herd proximity, quantity, movement and management of livestock creating opportunities for the introduction, transmission and establishment of FMD. The World Organisation for Animal Health (WOAH) estimates that FMD circulates in 77% of the world’s global livestock population, occurring in Africa, Asia and a part of South America (4). Although FMD is primarily documented in domestic livestock, FMDV has been reported to cause disease in over 70 wildlife species (5). Impalas (*Aepyceros melampus*) in close proximity with buffalo herds can become infected, develop clinical signs, and infect susceptible livestock (6, 7). However, only African buffalo (*Syncerus caffer*) is known to play an important role in maintaining FMD SAT-serotypes (8–10).

FMD outbreaks have also been documented in non-cloven-hoofed as well as hoofed wildlife species (11). For example, there are reports of natural FMDV infection in the, Arabian Oryx (*Oryx Leucoryx*), Impala (*Aepyceros melampus*), kudu (*Tragelaphus strepsiceros*) Asiatic elephant (*Elephas maximus*), capybara (*Hydrochoerus hydrochaeris*), European hedgehog (*Erinaceus europaeus*), eastern grey kangaroo (*Macropus giganteus*), domestic dog (*Canis lupus familiaris*) among others (6, 12–17). However, it should be noted that most of these outbreaks occurred in captive wild animals who, although they live under human supervision, are not confined and are instead part of wildlife parks adjacent to livestock. Experimental infection with FMDV has also been demonstrated in various atypical animals including armadillo, birds, cats, dogs, marsupials, moles, monotremes, primates, reptiles and rodents (17). As FMD research is primarily livestock-orientated, its epidemiological manifestation in atypical species is poorly documented or understood and warrants attention.

There have been three reports of FMD in brown bear (*Ursus arctos*), Asiatic black bears (ABB) (*Ursus thibetanus*) and Malayan sun bear (MSB) (*Helarctos*) (18–20). However, except for the manuscript by Officer, FMDV could not be isolated. The identification of FMDV in these papers, is based on clinical signs, which appear to be severe and limited to the footpads (19), and linked to FMDV outbreaks in surrounding area.

The Vietnam Bear Rescue Centre (VBRC) reported a period of 15 days in August 2011 when 13 Asiatic black bears and one Malayan sun bear displayed signs of lethargy and developed footpad vesicles, which were consistent with clinical signs of FMD and warranted further investigation. A minority of the bears in contact with these infected bears remained asymptomatic, while signs in the infected bears resolved by the end of September (20). Additionally, clinical records indicated FMD cases had occurred in a second location earlier in 2011. This provided an ideal opportunity for serological analysis to look for evidence of FMDV circulation in asymptomatic bears. Through virus isolation and sequencing FMDV serotype O was confirmed to be responsible for the outbreak, and it was hypothesised that the source of infection was a closely related isolate that may have been circulating in rural Vietnamese pigs at the time, although direct evidence is lacking since the FMD outbreak reporting rate in the country is low (20). Building on the previous study, the aims of this investigation were to (i) undertake a descriptive epidemiological analysis of the outbreak which includes the estimation of epidemiological parameters, (ii) identify risk factors and (iii) determine the nature of the antibody response, including longevity, in these unusual host species. Of particular interest was to elucidate persistence and differences in antibody titres between bear species, cubs and adult bears, and antibody responses in those bears that did not exhibit clinical signs of FMD.

## 2 Materials and methods

**Figure 1:**
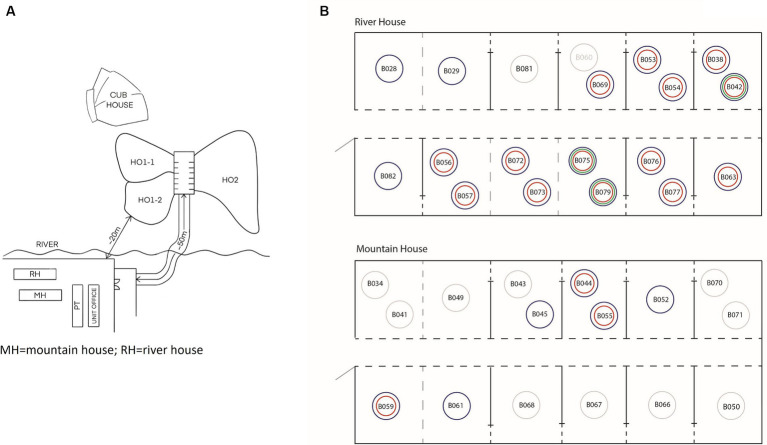
Enclosure layout of Vietnam Bear Rescue Centre **(A)**. Layout and location of bears in Mountain House and River House in 2011 **(B)**. Bears IDs are shown within circles; with grey ID indicating a deceased bear. A red circle indicates bears that displayed FMD clinical signs; a green circle indicates bears with positive test result for FMDV by virus isolation, PCR or ELISA [in the previous study (20)]; a blue circle indicates bears with serum sample/s collected and tested in the current study; a grey circle indicates bears with no clinical signs or testing. A grey dashed line indicates bears in neighbouring dens are integrated and have use of both dens; a solid line with black dashed portion indicates neighbouring bears are not integrated but have contact through den bars; a long horizontal black dashed line indicates den bars facing the central corridor. Figure is not to scale and for demonstrative purposes only.

In 2011, the VBRC housed 79 bears: 73 Asiatic black bears and 6 Malayan sun bears. The age range of the bears was 0.5–15 years, and time residing in the sanctuary ranged between 1–48 months. The centre comprised 5 houses: the Mountain House (17 bears), River House (19 bears), cub house (2 bears), H01-1 (5 bears), H01-2 (15 bears) and H02 (21 bears) (Figure 1). Bear-keeping staff lived in the nearby rural area and changed boots upon arrival, walked through 2% bleach footbaths when entering and leaving a bear house, and did not work between houses.

### 2.1 Study design

The study was performed following two FMDV outbreaks among captive bears at Animal Asia’s Vietnam Bear Rescue Centre (VBRC) in Tam Dao National Park, Vietnam. A total of 77 sera samples from 23 bears (22 Asiatic black bears and one Malayan sun bear) were tested during varying time points pre- and post-outbreak (between 24 months prior to 55 months post development of clinical signs), in order to characterise FMD antibody responses over time.

### 2.2 Animal selection

Bears were included in the analysis if they either displayed clinical signs of FMD, or if they did not display clinical signs but were in direct contact with those that did, for example, through open bars in adjacent dens. Bears included in the analysis were from two different houses: Mountain House and River House (Figure 1). Slides between dens could be opened and bears moved between dens for cleaning or enrichment sessions. The Asiatic black bears ranged from cubs through to adults and included both males and females. The one Malayan sun bear sampled was a female yearling exhibiting clinical signs of FMD. Blood samples were opportunistically collected during routine health checks; therefore time-points vary between subjects. Both pre- and post-outbreak samples were available for analysis.

### 2.3 Sampling and serology

After whole blood samples were aseptically collected from the jugular vein, sera were separated and frozen at −80°C. The serum samples were transported to the FAO World Reference Laboratory for FMD (WRLFMD) at The Pirbright Institute (TPI) under CITES export permit number 17VN2492N/CT-KL and importer permit number 556181/02. These sera were shipped on ice, where they were heat inactivated in a water bath at 56°C for 30 min and stored at 4.5°C ± 3.5°C prior to serological testing. The samples were tested by non-structural protein (NSP) ELISA using the commercially available PrioCHECK^®^ FMDV NS kit (Lot No.: F161002LA) from Prionics Lelystad B.V., following the technical insert version 1.0_e, which was adapted to allow testing using two wells per sample. Percent inhibitions greater than or equal to 50% were considered positive. Further analysis was conducted through the well-established Virus Neutralisation Test (VNT) method using IB-RS-2 cells and testing for anti FMDV neutralising antibodies against an isolate from the O/ME-SA/PanAsia lineage: O VIT 28/2014 (21). The neutralisation titre is the reciprocal of the serum dilution where 50% of the wells are protective at a virus dose of 100TCID_50_ (32 TCID_50−_320 TCID_50_). Neutralisation titres above or equal to 45 were considered positive while values less than 16 were considered negative. Neutralisation titres in-between were considered inconclusive.

### 2.4 Data management

All data were collated and stored in Excel. Data included the house layouts, characteristics of all animals (*n* = 79), description of clinical signs, dates of onset/regression of FMD clinical signs (*n* = 17), dates of sera collection and FMDV-antibody test results measured by non-structural proteins (NSP) and virus neutralisation tests (VNT) (*n* = 23).

### 2.5 Statistical analysis

**Figure 2:**
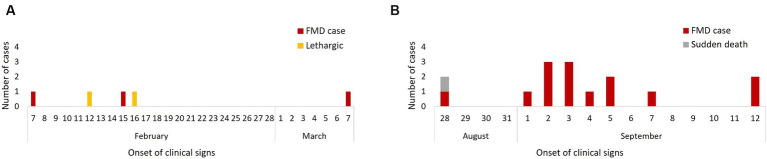
Epidemic curves of cases of FMD by date of clinical signs onset in the 2011 FMD outbreak: February–March (*n* = 3) **(A)** and August–September (*n* = 17) **(B)**.

Descriptive statistics were conducted. Temporal and spatio-temporal patterns of each outbreak were described by producing epidemic curves (Figure 2) and describing case-data and likely transmission chains. Clinical signs of FMD cases were described and tabulated.

The outbreak specific attack rates of FMD were estimated by dividing the number of bears developing clinical signs of FMD by bears present in the house where each phase of the outbreak happened. Given the sanctuary lay-out and geographical separation with other houses (Figure 1) only bears present in the affected houses were considered. The attack rates were used to calculate the basic reproduction numbers (*R*_0_) for the two outbreaks.

Multivariable analysis using a binomial logistic regression model was conducted to determine the extent to which sex and age were associated with FMD status (i.e., showing/not showing FMD clinical signs). Only bears housed in Mountain House or River House at the start of each outbreak were considered. Sex was analysed as a categorical variable whereas age was analysed as continuous. Differences between species were not considered due to lack of variability (only 1 Malayan sun bear). A *p*-value of <0.05 was deemed to indicate statistical significance. Likelihood ratio test was used to assess which model fit the data best (including both variables vs. only one of them).

The time to detection of FMDV-antibodies (seroconversion) was analysed by plotting overall and bear-specific VNT titres and NSP percentage inhibition against months pre- and post-onset of clinical signs or outbreak start for FMD cases and non-cases, respectively. McNemar’s chi-squared test for paired data and kappa statistics were used to assess whether there was a statistically significant difference in the proportion of positives between NSP and VNT tests, and to what extent they were correlated.

All statistical analyses were conducted in R.4.2.0 (R Core Team, 2022) using packages epiR (function epi.kappa) (22), lme4 (function glm) (23) and R0 (function estimate.R) (24).

## 3 Results

Bears displaying clinical signs consistent with FMD were recorded in the Mountain House (*n* = 3; February–March 2011) and River House (*n* = 14; August–September 2011). Both houses consisted of rows of indoor dens only along a central corridor, and bears in neighbouring dens could have physical contact through metal bars. Each den was separated by a sliding metal bar door, the bears could be moved between adjacent dens during cleaning and enrichment set-up sessions (Figure 1). The first outbreak exclusively affected the Mountain House and started on February 7 (day 1), when a female Asiatic black bear (B055) demonstrated signs of FMD, after which another adult Asiatic black bear (B044) in the same den was found to have signs on day 9 and a final case (B059) developed clinical signs on day 29. Two adult bears (B043, B070) in in the same house demonstrated lethargy on day 6 and 9 of the outbreak but showed no other clinical signs of FMD. No diagnostic testing was carried out during this outbreak.

The second outbreak affected only the River House with the first case reported on August 28 (day 1) when an adult male Asiatic black bear (B069) showed clinical FMD and a second bear (B060) in the same den suddenly died with non-specific clinical signs (lethargy and inappetence only). Post-mortem examination was undertaken and there were no gross signs of FMD. Samples had not been taken from this bear and was not considered in the rest of the analysis. Four days later (day 5) an adult female bear (B054) in a neighbouring den developed FMD signs followed by her den-mate (B053) on day 7. Simultaneously, from days 6–8 all 6 animals (5 juveniles – B056, B057, B072, B073, B075, 1 adult – B079) in a den across the corridor from the index case developed signs of FMD, followed by 2 cubs in the adjacent den on day 9. Two days later (day 11) a Malayan sun bear in the den next to the cubs showed clinical FMD, followed by the 2 final cases in adult Asiatic black bears (B038, B042) on days 15 and 16. Diagnostic work ups were performed on three affected bears (B079, B075, and B042), with FMDV positive RT-PCR results recorded for all three cases, and isolation of FMDV from B079 and B042 (ref the original paper). Faeces from B063 and samples opportunistically collected 10 days after the onset of signs from B038 tested negative. Epidemic curves suggested a propagated and a point-source outbreak (Figures 2A,B).

The attack rate of FMD during the first outbreak was 18.75% (95% confidence interval (CI): 4.97–46.3%). The second outbreak had a higher attack rate of 77.77% (95% CI: 51.92–92.63%). Based on these attack rates the basic reproduction number (*R*_0_) for the Mountain House outbreak was 1.11 (95% CI: 0.99–1.27), while for the River House outbreak it was 1.93 (95% CI, 1.50–3.61).

All affected bears (*n* = 17) developed blister-like lesions on all their foot-pads, presenting as noticeably more severe in younger animals. Of all cases, 35.29% (*n* = 6) and 11.76% (*n* = 2) also developed blisters on their nose and lips, respectively (Table 1). Blisters on the nose appeared 2–11 days (mean = 4.83 days) after blisters on footpads. Fifteen bears exhibited reduced activity for periods ranging from 1–12 days (mean = 5.53 days, 95% CI: 3.85–7.21), with the remaining 2 cases, the cubs, showing no change in activity. Nine cases (52.94%) had appetite reductions lasting from 1–4 days post-onset of clinical signs (mean = 1.72 days, 95% CI: 0.84–2.61). Footpad lesions took 16–17 days to fully heal (based on 4 observations) and sloughed between 2–21 days (based on 11 observations). From all cases, 14 animals (82.35%) were administered analgesia (tramadol, *n =* 2; meloxicam, *n* = 12), antibiotics (cephalexin, *n* = 12; amoxicillin clavulanate, *n* = 1) and/or a neuroleptic agent (acepromazine, *n* = 1).

**Table 1:** Summary of clinical signs of FMD cases in bears from both outbreaks (*n* = 17) in Vietnam Bear Rescue Centre, 2011.

Clinical sign^1^	Number of days	FMD cases (*n* = 17)	
		*n*	%
Blisters on foot pads		17	100
Blisters on nose		6	35.3
Blisters on lips		2	11.8
Reduced activity (number of days post onset of signs)	0	2	11.8
	1–5	7	41.2
	6–10	6	35.3
	11+	2	11.8
Reduced appetite (number days post onset of signs)	0	8	47.1
	1	5	29.4
	2–4	4	25.5

FMD, foot-and-mouth disease.

1 Clinical signs were observed and reported by staff working at each house.

Overall, three out of 16 (18.8%) and 14 out of 18 (77.8%) bears in the affected houses developed clinical signs of FMD in the first and second outbreak, respectively (Table 2). All cases affected Asiatic black bears, with the exception of one Malayan sun bear which was the only Malayan sun bear housed in an FMD-affected house. In both outbreaks, a slightly greater proportion of females contracted FMD compared to males. Younger animals appeared to have an increased incidence of FMD (mean age of cases = 3.9 years), with all cubs (*n* = 2) and 85.7% (*n* = 6) of yearlings becoming infected as opposed to none of the adults aged 10+ years and older (*n* = 2) in the River House outbreak (Table 2).

**Table 2:** Demographic characteristics of FMD cases in the Mountain House (February–March 2011; *n* = 3) and River House (August–September 2011; *n* = 14) in the Vietnam Bear Rescue Centre.

Characteristic	Category	Mountain House outbreak (February–March 2011)				River House outbreak (August–September 2011)			
		Subjects		FMD Cases		Subjects		FMD Cases	
		*n*	%^1^	*n*	%^2^	*n*	%	*n*	%
**Total**		**16**	**100**	**3**	**18.75**	**18**	**100**	**14**	**77.78**
Species	Asian black bear	16	100	3	18.8	17	94.4	13	76.5
	Malayan sun bear	–	–	–	–	1	5.6	1	100
Sex	Male	9	56.3	1	11.1	7	38.9	5	71.4
	Female	7	43.8	2	28.6	11	61.1	9	81.8
Age group (years)	Cub (0)	0	0	0	0	2	11.1	2	100
	Yearling (1)	0	0	0	0	7	38.9	6	85.7
	Adult (2–9)	8	50.0	2	25.0	7	38.9	6	85.7
	Adult (10+)	8	50.0	1	12.5	2	11.1	0	0
Time in sanctuary (months)	0–6	0	0	0	0	5	27.8	3	60.0
	7–12	11	68.8	3	27.3	5	27.8	5	100
	13–24	5	31.3	0	0	8	44.4	6	75.0

1 Indicates percentage of animals in the house in the category (row %).

2 Indicates percentage of animals in category with FMD (column %).

The odds of showing clinical signs were higher in females [adjusted odds ratio (AOR): 11.58; 95% CI: 1.29–293.6; *p* = 0.06] and decreased with age (AOR: 0.51; 95% CI: 0.30–0.73; *p* = 0.003).

### 3.1 Relationship between seroconversion and clinical signs

**Figure 3:**
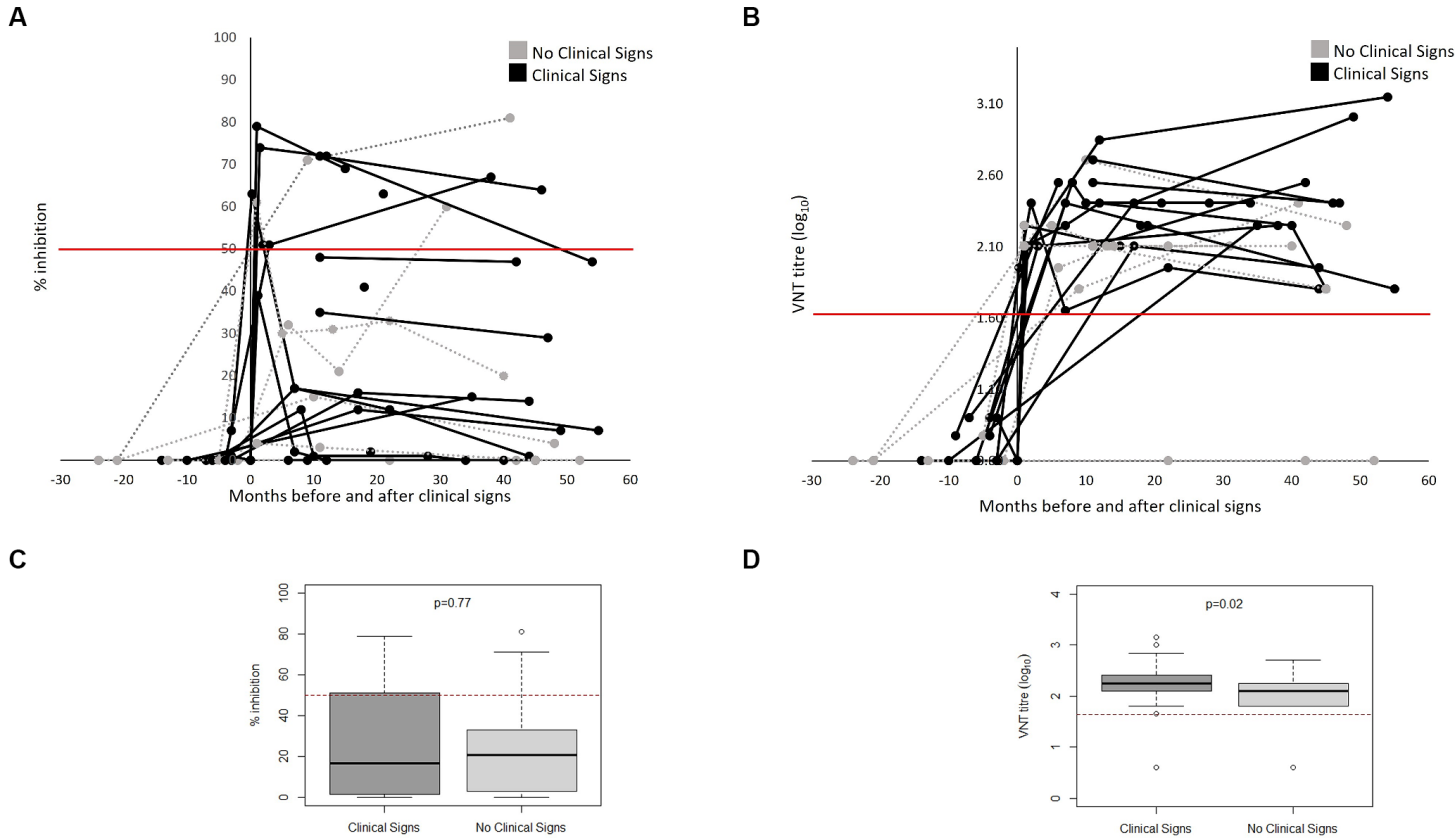
FMDV-antibodies in bears FMD cases (*n* = 17) and non-cases (*n* = 6) from both outbreaks as measured by NSP **(A)** and VNT **(B)** antibody tests over months pre- and post-onset of clinical signs (for cases) or pre- and post-outbreak (for non-cases) in Vietnam Bear Rescue Centre. FMD cases were defined as bears demonstrating clinical signs of FMD. Clinical signs were first observed at x = 0. For non-cases, x = 0 indicates the start of the outbreak. Antibody tests were deemed positive if VNT titre >45 and NSP percent inhibition ≥50, represented with a red line. Boxplots comparing titles as measured by NSP **(C)** and VNT **(D)** of clinical and non-clinical bears considering results from x = 0 onwards.

Figure 3 shows the FMDV-specific antibody levels in FMD cases (defined as presence of clinical signs) (*n* = 17) and non-cases (defined as absence of clinical signs) (*n* = 6), from both outbreaks, measured by VNT and NSP ELISA over time relative to the date of onset of clinical signs (for cases) or start of the outbreak (for non-cases). Not all bears had samples collected (see Supplementary Table S1), and therefore only bears with samples collected are presented in Figure 3. All cases tested positive to VNT following onset of clinical signs and remained positive for the rest of the follow up period. Two animals were sampled only 1 day post onset of clinical signs and tested negative to VNT in this sampling; however, they were positive in the following sampling. Only 6 out of 17 cases tested positive to NSP following the development of clinical signs. Of the 6 non-cases tested, 5 seroconverted to VNT, 3 to NSP post-outbreak and one remained negative to both assays. There was a significant difference and only slight agreement between NSP and VNT for samples from cases (kappa statistic 0.04) and fair agreement for those from non-cases (kappa statistic 0.23) (Table 3). All samples collected from animals before the first outbreak tested negative to both tests (NSP and VNT). All bears that seroconvert in the River House were after the onset of the second outbreak.

**Table 3:** Concordance between number of positive and negative results using NSP and VNT tests.

	VNT		*p*-value†	Kappa statistic
	Number of negative	Number of positive		
*All samples* (*n* = 77)				
**NSP**				
Number of negative Number of positive	24 0	38 15	<0.001	0.19
*Samples from animals with clinical signs* (*n* = 40)				
**NSP**				
Number of negative Number of positive	2* 0	27 11	<0.001	0.04
*Samples from animals without clinical signs* (*n* = 26)				
**NSP**				
Number of negative Number of positive	11 0	11 4	0.002	0.23

†McNemar test.A significant test suggests strong disagreement between the tests.*These bears were tested 1 day after clinical signs onset.

## 4 Discussion

This study provides a description of two incursions of an FMD outbreak in captive bears. Understanding the evolution of the outbreak, transmission dynamics and clinical presentation in bears provides essential information on the potential impacts of future FMD outbreaks, informing disease control measures (25) and the design of bear sanctuaries and their biosecurity protocols.

The attack rate of FMD during the first outbreak was 18.75% while the second outbreak had a higher attack rate of 77.77%. The corresponding basic reproduction numbers (*R*_0_) were 1.11 and 1.93, respectively, which are close to the epidemic threshold at *R*_0_ = 1, especially in the first phase of the outbreak. They are also much lower than is reported for outbreaks in typical hosts (*R*_0_ ~ 10–20) such as cattle in previously naïve populations (26) or African buffalo under experimental conditions (27), but similar to values in cattle in endemic populations (*R*_0_ ~ 1.26–2.52) (25). This could reflect lower transmissibility of FMDV in bears but could also reflect the reduced opportunity for contact between individuals because of the way the bears were housed.

After adjusting for sex, there was a strong association (*p* = 0.003) between age and the presentation of FMD clinical signs, with decreased odds of FMD signs per year of age (AOR = 0.51). In addition, younger animals showed more severe blister-like lesions. These findings are in line with current literature in other species (28, 29). It is plausible that the higher proportion of younger bears kept in River House at the time of the second outbreak contributed to the increased cases and level of transmission in this incursion. Additionally, despite the River House enforcing control measures on day 6 of the outbreak, all animals tested from the River House were seropositive. This highlights the need to take immediate action to control the spread of the virus, especially as it is still unknown whether bears are infectious prior to the onset of clinical signs.

Following the second outbreak, biosecurity protocols were reviewed and updated including requiring the staff to change clothes before entering and upon exiting the sanctuary, as some of the staff keep livestock and feed their animals before going to work. All personnel must now also go through Virkon footbaths when entering and exiting bear housing areas and all guests on site must also follow the biosecurity change in/change out policy if they enter bear housing areas during their visit. In addition, all bears are placed into quarantine for 30-45 days when they arrive to allow for close observation of any health issues and to treat if necessary, as well as allowing rapport building with staff to gain trust in the bears. Since the biosecurity measures were strengthened no outbreaks have been reported in the centre even though FMDV is still circulating in rural areas nearby.

Serological analysis found that both symptomatic and asymptomatic infection with FMD conferred rapid and long-lasting antibody responses in bears, which supports current literature (7, 30, 31). This study highlights the general importance of collecting sera routinely, not just during infectious disease outbreaks, but also for retrospective analyses to better understand transmission dynamics.

All FMD cases seroconverted post-outbreak by VNT and 35.3% (*n* = 6/17) when using NSP. A similar pattern was observed in non-cases. Although occasional disagreement between the NSP and VNT could be expected as the tests detect different subsets of antibodies, we observed significant differences and only weak concordance between them. Considering just bears that developed clinical signs, the sensitivity of VNT was 100% (CI 89–100%) while the sensitivity of NSP was 27% (CI 15–44%). All bears that seroconverted to VNT remained sero-positive until the end of the follow up period, which in some bears was up to 4.5 years after infection. We note that the NSP ELISA kit is not validated for the use in bears and therefor the precise cut-off for positive samples is not known. Some samples showed toxicity in the VNT which could have caused false negatives in the NSP ELISA as these samples were not titrated. In addition, a limitation of this study is the irregular intervals at which serum samples were collected, suggesting that initial antibody responses may have been missed. However, given samples were derived from large carnivores with serum sample collection requiring general anaesthesia, this limitation is attributed to the nature of the study population. For similar reasons, clinical signs of FMD were observed only through protected contact conscious examinations through den bars, not through hands-on close examination, meaning less severe signs may have been missed, leading to differential misclassification and an underestimation of the effect estimates.

Animals can contract FMDV through inhalation, ingestion, or direct contact with viral particles. Additionally, FMDV can remain viable in the environment for up to 2 weeks (32–34). The environmental persistence of FMDV could explain why the first outbreak appeared to have a propagated source. Moreover, as the infectiousness of subclinical FMD cases is not well known, asymptomatic cases which seroconverted post-outbreak may have played a role in FMD transmission within the house.

Further research on the contribution of subclinical FMD in bears to disease transmission, the duration of infectiousness, and the level of antibody response required to confer FMDV-immunity warrants attention. Previous research reports differences between inter- and intra-species FMD transmissibility, such as increased cattle-cattle transmission compared to cattle-sheep or sheep-sheep (35, 36). The role of bears in inter-species FMD transmission therefore comprises an important area of future research, particularly when considering the potential of wildlife-livestock interactions and introduction to rescue centres. Results from this outbreak investigation suggest that bears were infected by an external source on two separate occasions, and by adhering to strict biosecurity and quarantine protocols, bears in rescue centres or zoos can be kept FMD free in areas where FMD is endemic.

## 5 Conclusion

FMDV can cause clinical and subclinical disease in bears, both of which appear to confer rapid and long-lasting antibody responses. This study provides initial epidemiological parameters of FMD in bears and suggests that bears were accidental hosts in this outbreak, and FMD outbreaks can be prevented if strict biosecurity protocols are put in place.

## Data availability statement

The original contributions presented in the study are included in the article/Supplementary material, further inquiries can be directed to the corresponding author.

## Ethics statement

The animal study was approved by Social Science Research Ethical Review Board (SSRERB) (URN SR2020-0176), Royal Veterinary College, UK. The study was conducted in accordance with the local legislation and institutional requirements.

## Author contributions

AL: Conceptualization, Data curation, Visualization, Writing – original draft, Writing – review & editing. HB: Methodology, Writing – review & editing. RS: Writing – original draft, Writing – review & editing. RJ: Data curation, Formal analysis, Writing – review & editing. JM: Methodology, Project administration, Writing – review & editing. MW: Supervision, Writing – review & editing. DK: Conceptualization, Funding acquisition, Resources, Writing – review & editing. SG: Formal analysis, Supervision, Writing – review & editing. GL: Conceptualization, Formal analysis, Writing – original draft, Writing – review & editing. KO: Conceptualization, Funding acquisition, Methodology, Writing – original draft, Writing – review & editing.
