# Supplementary material for: Epidemiological investigation of foot-and-mouth disease outbreaks in a Vietnamese bear rescue centre
Source: Front Vet Sci. 2024 Jun 17;11:1389029. doi: 10.3389/fvets.2024.1389029 (PMC11215046; doi:10.3389/fvets.2024.1389029)
Supplement: Supplementary file 1 [file Table_1.DOCX]

Table S1. Bear characteristics, location and samples collected.

| **Bear ID** | **Species** | **Sex** | **Age**  **(in years)** | **Clinical signs** | **Samples collected** | **Samples collected –**  **Months in relation to onset of clinical signs (for cases) and beginning of the outbreak (for no-cases)** | **House** |
| --- | --- | --- | --- | --- | --- | --- | --- |
| B044 | ABB | Female | 10 | Yes | Yes | -7^a,b^, 17^a,d^, 49^a,d^ | Mountain |
| B045 | ABB | Male | 15 | No | Yes | -13^a,b^, -6^a,b^, 22^a,b^, 42^a,b^, 52^a,b^ | Mountain |
| B052 | ABB | Female | 10 | No | Yes | -2^a,b^, 6^a,d^, 14^a,d^, 31^c,d^ | Mountain |
| B055 | ABB | Female | 7 | Yes | Yes | -3^a,b^, 17^a,d^, 44^a,d^ | Mountain |
| B059 | ABB | Male | 4 | Yes | Yes | -4^a,b^, 3^c,b^, 38 ^c,b^ | Mountain |
| B061 | ABB | Male | 10 | No | Yes | -5^a,b^, 1^c,d^, 5^a,d^, 13^a,d^, 22^a,d^, 40^a,d^ | Mountain |
| B034 | ABB | Female | 10 | No | No | - | Mountain |
| B041 | ABB | Male | 7 | No | No | - | Mountain |
| B043 | ABB | Male | 10 | No | No | - | Mountain |
| B049 | ABB | Female | 8 | No | No | - | Mountain |
| B050 | ABB | Male | 10 | No | No | - | Mountain |
| B066 | ABB | Female | 9 | No | No | - | Mountain |
| B067 | ABB | Male | 9 | No | No | - | Mountain |
| B068 | ABB | Female | 10 | No | No | - | Mountain |
| B070 | ABB | Male | 8 | No | No | - | Mountain |
| B071 | ABB | Male | 9 | No | No | - | Mountain |
| B028 | ABB | Female | 10 | No | Yes | -24^a,b^, -21^a,b^, 9^c,d^, 41^c,d^ | River |
| B029 | ABB | Female | 10 | No | Yes | -24 ^a,b^, -21^a,b^, 10^a,d^, 48^a,d^ | River |
| B038 (B5)* | ABB | Female | 7 | Yes | Yes | -3^a,b^, 1^c,d^, 2^c,d^, 7^a,d^, 22^a,d^, 44^a,d^ | River |
| B042 (B4)* | ABB | Female | 8 | Yes | Yes | -14^a,b^, 0^a,b^, 1^c,d^, 15^c,d^ | River |
| B053 | ABB | Female | 5 | Yes | Yes | -6^a,b^, 8^a,d^, 10^a,d^, 28^a,d^, 34^a,d^ | River |
| B054 | ABB | Female | 7 | Yes | Yes | -10^a,b^, 35^a,d^ | River |
| B056 | ABB | Female | 1 | Yes | Yes | 18^a,d^ | River |
| B057 | ABB | Male | 1 | Yes | Yes | 11^a,d^, 42^a,d^ | River |
| B063 (B3)* | MSB | Female | 1 | Yes | Yes | -9^a,b^, 6^a,d^ | River |
| B069 | ABB | Male | 8 | Yes | Yes | -4^a,b^, 7^a,d^, 55^a,d^ | River |
| B072 | ABB | Male | 1 | Yes | Yes | 11^c,d^, 46^c,d^ | River |
| B073 | ABB | Male | 1 | Yes | Yes | 11^a,d^, 47^a,d^ | River |
| B075 (B2)* | ABB | Female | 1 | Yes | Yes | 0^a,b^, 1^c,d^, 12^c,d^, 54^a,d^ | River |
| B076 | ABB | Female | 0 | Yes | Yes | 21^c,d^ | River |
| B077 | ABB | Male | 0 | Yes | Yes | 19^a,d^ | River |
| B079 (B1)* | ABB | Female | 4 | Yes | Yes | -3^a,b^, 0^a,b^, 1^a,d^, 7^a,d^, 12^a,d^, 40^a,d^, 45^a,d^ | River |
| B082 | ABB | Male | 1 | No | Yes | 1^a,d^, 11^a,d^, 45^a,d^ | River |
| B081 | ABB | Male | 7 | No | No | - | River |

*Tested in Officer et al (2011), in brackets the IDs from previous study.

ABB= Asiatic black bear, MSB= Malayan sun bear

^a^ Negative to NSP

^b^ Negative to VNT

^c^ Positive to NSP

^d^ Positive to VNT
